# The roles of DNA methylation on the promotor of the Epstein–Barr virus (EBV) gene and the genome in patients with EBV-associated diseases

**DOI:** 10.1007/s00253-022-12029-3

**Published:** 2022-06-28

**Authors:** Linlin Zhang, Ran Wang, Zhengde Xie

**Affiliations:** 1 Present Address: Beijing Key Laboratory of Pediatric Respiratory Infectious Diseases, Key Laboratory of Major Diseases in Children, Ministry of Education, National Clinical Research Center for Respiratory Diseases, Beijing Pediatric Research Institute, Beijing Children’s Hospital, Capital Medical University, National Center for Children’s Health, Beijing, 100045 China; 2 Research Unit of Critical Infection in Children, Chinese Academy of Medical Sciences, Beijing, 2019RU016 China

**Keywords:** Epstein–Barr virus, Epigenetics, DNA methylation, EBV-associated diseases

## Abstract

**Abstract:**

Epstein–Barr virus (EBV) is an oncogenic virus that is closely associated with several malignant and lymphoproliferative diseases. Studies have shown that the typical characteristic of EBV-associated diseases is aberrant methylation of viral DNA and the host genome. EBV gene methylation helps EBV escape from immune monitoring and persist in host cells. EBV controls viral gene promoter methylation by hijacking host epigenetic machinery to regulate the expression of viral genes. EBV proteins also interact with host epigenetic regulatory factors to mediate the methylation of the host’s important tumour suppressor gene promoters, thereby participating in the occurrence of tumorigenesis. Since epigenetic modifications, including DNA methylation, are reversible in nature, drugs that target DNA methylation can be developed for epigenetic therapy against EBV-associated tumours. Various methylation modes in the host and EBV genomes may also be of diagnostic and prognostic value. This review summarizes the regulatory roles of DNA methylation on the promotor of EBV gene and host genome in EBV-associated diseases, proposes the application prospect of DNA methylation in early clinical diagnosis and treatment, and provides insight into methylation-based strategies against EBV-associated diseases.

**Key points:**

*• Methylation of both the host and EBV genomes plays an important role in EBV-associated* *diseases.*

*• The functions of methylation of the host and EBV genomes in the occurrence and development of EBV-associated diseases are diverse*.

*• Methylation may be a therapeutic target or biomarker in EBV-associated diseases*.

## Introduction

Epstein–Barr virus (EBV) was originally discovered by Michael Epstein and Yvonne Barr in a cultured lymphoma cell line from African Burkitt’s lymphoma (BL) patients through electron microscopy in 1964, and it was the first identified human tumor virus (Epstein et al. 1964). EBV belongs to the γ-herpesvirus family and is also known as human herpes virus type 4. The DNA genome of EBV is estimated to be 172 kb, and it encodes more than 90 genes (Odumade et al. 2011). Humans are susceptible to developing EBV infections, and EBV can establish lifelong latent infection in more than 90% of the adult population (Kwok and Chiang 2016). EBV is mainly transmitted through saliva and transfusions. The primary infection with EBV usually occurs by contact with saliva from infected individuals, and then, the virus enters epithelial cells of the oropharynx, where the virus is amplified through lytic replication and subsequently infects B cells and oropharyngeal epithelial cells (Bu et al. 2019). EBV shows obvious tropism to B lymphocytes and transforms them into continuously proliferating lymphoblastoid cell lines (LCLs). Ultimately, latent infection is established with the expression of viral latent genes and without the production of infectious virus (Hutt-Fletcher 2007).

Most EBV primary infections occur during childhood and adolescence and are mostly asymptomatic, whereas some patients infected with EBV manifest infectious mononucleosis (IM) (Bu et al. 2019), which is benign and self-limited. The course ranges from 2 to 3 weeks, and the majority of patients recover well; however, a few individuals manifest with chronic recurrent IM-like symptoms over a long period of time, accompanied by an unusual pattern of antibodies against EBV and high EBV DNA load in the peripheral blood, which is referred as chronic active EBV infection (CAEBV). The pathogenesis of CAEBV has not yet been clarified, and the prognosis is unsatisfactory and often results in high mortality rates. Unfortunately, there is still no definite therapy for CAEBV (Cohen et al. 2011). In addition, EBV, as an important oncogenic virus, is causally associated with various malignancies (Zhang et al. 2020), including epithelioid malignancies such as nasopharyngeal carcinoma (NPC), EBV-associated gastric carcinoma (EBVaGC), breast cancer (BC) and lymphoid malignancies such as BL, Hodgkin’s lymphoma (HL), and T/natural killer (NK) cell lymphoma (Ai and Xie 2018; Tsao et al. 2017). Although EBV has been identified and studied for over 50 years, its complex mechanisms have not been fully elucidated. At present, plenty of evidence shows that aberrant epigenetics are involved in EBV-associated diseases.

Epigenetic modifications are heritable and affect gene expression without altering DNA sequences (Jones and Baylin 2007; Sharma et al. 2010). Epigenetic modifications include but may not be limited to DNA methylation, chromatin remodeling, nucleosome positioning, and histone modifications, with DNA methylation being the most commonly studied epigenetic modification in humans (Leong and Lung 2021). DNA methylation is generated by the activity of DNA methyltransferases (DNMTs), which enzymatically transfer a methyl group from S-adenosyl methionine (SAM) to cytosine, mainly occurring at the fifth carbon atom of cytosine (5mC) in mammalian DNA (Jair et al. 2006). DNA methylation influences gene expression patterns without altering the corresponding DNA sequence, and it is a reversible and heritable epigenetic modification that is important in gene regulation and organism development. There are three major DNMTs for DNA methylation. DNMT1 is mainly responsible for the maintenance of DNA methylation by “copying” the cytosine-phosphate-guanine (CpG) methylation pattern from the parental strand onto the daughter strand during DNA replication. DNMT3a and DNMT3b generally perform *de novo* methylation of either unmethylated or hemimethylated DNA (Moore et al. 2013). DNA methylation occurs primarily at cytosines (C) in CpG-enriched regions called CpG islands (CGIs) (Belleau et al. 2018). CGIs are thought to be predominantly located in the promoter region of genes, and approximately 50% of the human genome in the 5′ end of the gene promoter region contains CGIs. In normal cells, CGIs located in the housekeeping gene promoter are protected from methylation and allow for transcription of the downstream gene, whereas those located in genomic sequences other than the promoter CpG island tend to be methylated. Approximately 70–80% of CpG sites in the mammalian cells are methylated, but both the CpG sites and their degrees of methylation are unevenly distributed in the genome (Esteller 2007; Leong and Lung 2021). However, aberrant DNA hypermethylation of CGIs in promoters (promoter hypermethylation) usually occurs in many cancers. The three main mechanisms of promoter hypermethylation are as follows. (i) Direct effect: DNA methylation can directly interfere with the binding of transcription factors to their recognition sites. (ii) Indirect effect: the methylation of gene regulatory sequence in the 5′ end binds to the methylated CpG sequence-binding proteins and prevents the transcription factors from forming transcriptional complexes with the gene. (iii) CpG methylation: moderately changes the structural properties of DNA and thereby affects transcription through regulation of the chromatin structure (Zhang et al. 2021).

When the virus persists during latent infection, most of the CGIs of early virus gene promoters are methylated, and the expression of virus genes is inhibited and maintains latent infection. For example, the promoter of the transcription initiation region of the bovine leukaemia virus is methylated under the action of the host DNMT3b, thereby directly inhibiting the binding of transcription factors, blocking viral transcription and maintaining virus latency (Pierard et al. 2010). The viral proteins VP26 and VP5 of herpes simplex virus 1 and the latent-related nuclear antigen encoded by Kaposi sarcoma-associated herpesvirus all bind to host DNMT3a to promote DNA methylation of their viral gene promoters (Rowles et al. 2015). Similarly, the X protein encoded by hepatitis B virus (HBV) can also upregulate host DNMT gene expression and promote the methylation of viral DNA and can lead to HBV latency in hepatocytes. In addition, the transcription activator *Tax* encoded by HBV downregulates the expression of DNMTs, resulting in virus reactivation (Vivekanandan et al. 2010). Methylation participates in genomic defense that resists viral infection and protects the human genome from a large number of repeated malignant effects. However, accumulating evidence has suggested that some viruses, including EBV, can selectively methylate and silence certain regions of their own genomes, especially when the viral gene expression of this region in the host cell is not required for the survival, establishment, and maintenance of the latent infection (Weber et al. 2019).

To address the importance of aberrant methylation in EBV-associated diseases, we summarized recent advances in EBV genome methylation characteristics during EBV latency states and lytic reactivation. The epigenetic profiles of DNA methylation have been extensively studied in several EBV-associated diseases, including EBVaGC, NPC, HL, and BL, and the mechanisms contributing to the unique epigenetic signatures have also been comprehensively summarized and compared. Recent knowledge on EBV methylation suggests its potential as a therapeutic target or biomarker in EBV-associated diseases.

## DNA methylation of the EBV genome

Once EBV infects host cells, it starts to induce a latent or lytic infection with diverse expressed genes (Ma et al. 2011). According to the latent genes expressed by EBV in host cells, EBV infection can be classified into four distinct latent infection statuses (0, I, II, and III) (Chiu and Sugden 2016). Only two noncoding RNAs called EBV-encoded RNA 1 (*EBER1*) and *EBER2* are expressed at latency 0. For example, EBV can latently infect memory B cells of healthy carriers. EBV infection in latency I expresses three latent genes in the host cell, EBV nuclear antigen 1 (*EBNA1*), *EBERs*, and *BamH I-A* rightward transcripts (*BARTs*); this includes BL and plasmablastic lymphoma (Navari et al. 2015). The EBV in latency II expresses four latent genes, *EBNA1*, *EBERs*, *BARTs*, and latent membrane protein (*LMPs*). For example, HL, CAEBV, peripheral T cell lymphoma, diffuse large B cell lymphoma, NPC, and EBVaGC are classified as type II latency diseases (Sakamoto et al. 2017; Tsao et al. 2017). Hosts with type III latency, for example, patients with IM or immunocompromised individuals and immortalized LCLs, express all latent genes, including six *EBNAs* (*EBNA1*, *EBNA2*, *EBNA3A*, *EBNA3B*, *EBNA3C*, and *EBNA-LP*), *EBERs*, *LMP1*, *LMP2A*, and *LMP2B* (Li et al. 2020b). Under certain conditions, such as the body having decreased immune function or certain physiochemical factors, EBV can be reactivated, and a large number of viral structural and regulatory genes can be expressed. The lytic replication cycle is characterized by the ordered and sequential expression of viral immediate-early (*BZLF1* and *BRLF1*), early (*BNLF1*, *BHRF1*, *BMRF1*, and *BARF1*), and late (*gp350*, *gp85*, and *gp110*) genes, which are regulated primarily at the level of transcription (McKenzie and El-Guindy 2015). Latent genes are selectively expressed based on the different latent phases of EBV in the infected cells. The strategy of selective expression of latent genes of EBV contributes to evading immune surveillance and establishing a lifelong persistent infection in the host (Ok et al. 2015).

Promoters encoding the EBNAs include the *BamH I W* promoter (Wp), *BamH I C* promoter (Cp), and *BamH I Q* promoter (Qp). Wp and Cp are the common transcription promoters of EBNAs that can initiate the transcriptional synthesis of all six EBNA mRNAs. However, Qp transcribes only EBNA1 mRNA and is not involved in the expression of other EBNAs. The transcriptional promoter of *LMP* (LMPp) is located in the *BamH I N* region, which is transactivated by EBNA2, and EBNA2 induces the expression of LMPs (Kartika et al. 2020). The EBV lytic cycle is initiated by the activation of two promoters, the *BZLF1* promoter (Zp) and *BRLF1* promoter (Rp), that direct the expression of the immediate-early genes *Zta* and *Rta*, which in turn activate the downstream lytic genes individually or coordinately (Jin et al. 2010; Tonoyan et al. 2020).

The effects of epigenetic modifications, especially DNA methylation, are involved in the whole process of EBV infection and promote the development of EBV-associated diseases. Some studies have reported that the methylation status of the EBV promoter is related to the activation of the latent promoter. The EBV genome is unmethylated but becomes heavily methylated during the latent stage of the virus cycle in infected cells (Germi et al. 2016). How EBV promoters obtain methylated cytosines is not yet clear; once established, this methylation pattern is maintained in proliferating and latently infected cells (Kalla et al. 2012). In addition to Qp, there is a good correlation between EBV latency promoter methylation and promoter activity (Guo and Gewurz 2022).

## DNA methylation of Wp

**Fig. 1:**
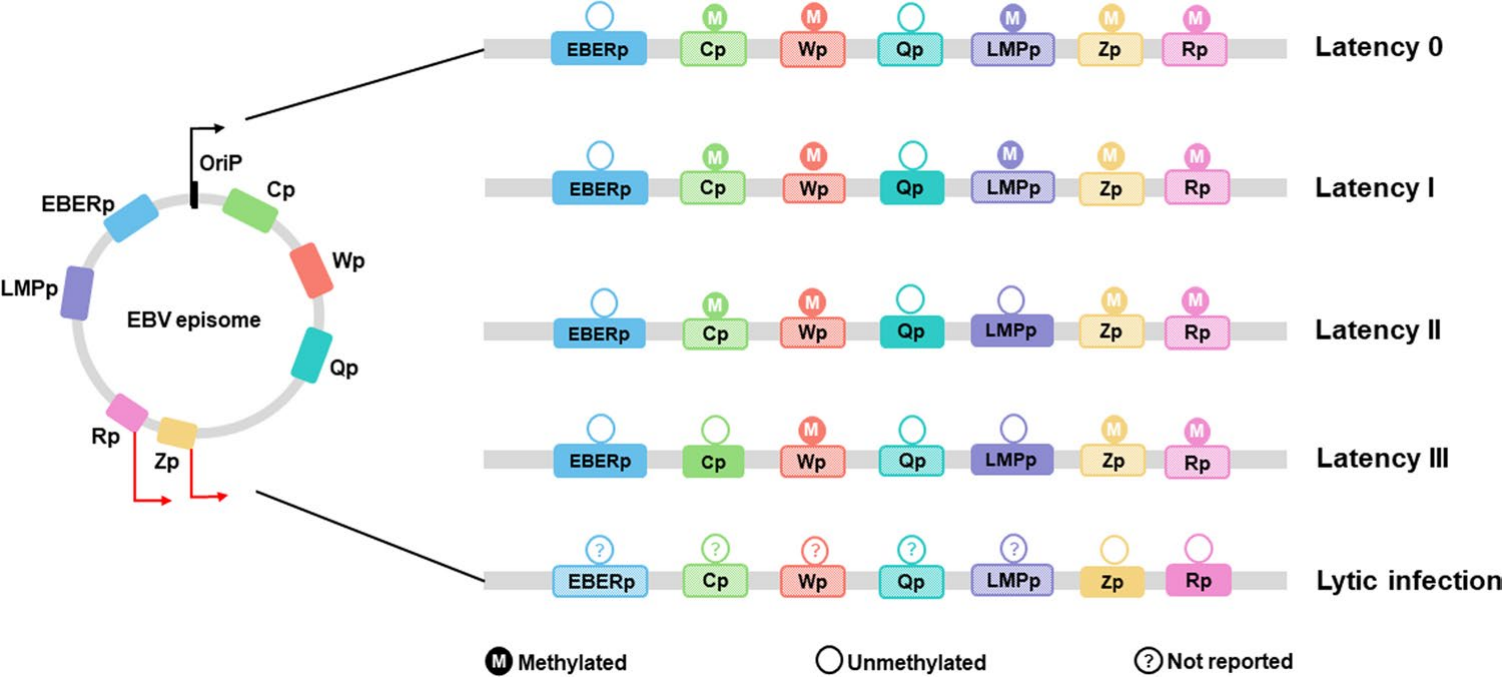
Epigenetic markers of EBV gene promoters. The EBV circular episome genome is shown with viral promoters (left). Promoter usage and expression during latent and lytic infection depend on DNA methylation (right). Symbols: arrow, directions of the encoding; solid squares, active promoters; dotted squares, silent promoters

Wp is the first EBV latency promoter used for transcription of the genes encoding EBNA2 and EBNA leader protein upon infection, and its regulatory elements are sensitive to CpG methylation. During early EBV infection, the EBV genome is packaged into virions and enters human cells; the entire genome is nonmethylated, Cp and Qp are in the off state, and Wp is in the on state (Belleau et al. 2018; Ling et al. 1993; Ponnusamy et al. 2019). Several days after EBV infection, the transcriptional activity of Wp closes, and Cp begins to activate and becomes the major latency promoter (Chelouah et al. 2018). The Cp of the LCL cell lines also shows a certain degree of methylation over time, but methylation cannot silence promoter transcription (Tao et al. 2002). In one study, sulfite genome sequencing was used to study Wp, and the transcriptional repression of viral genes was found to be closely associated with the methylation of Wp. Two kinds of cellular transcription factors, BSAP/Pax5 and CREB, are important in the transcriptional activity of Wp and are sensitive to methylation of DNA-binding sites in the promoter region. Wp methylation was detected seven days after infection, and almost all sites in the Wp region were methylated 18 days after infection (infectious virus particles showed extensive hypomethylation of the virus genome) (Chelouah et al. 2018). In the latency I program, Qp is the active promoter, while Wp and Cp are silent promoters (Fig. 1). When BL cells in latency I are transformed into latency III in vitro, Cp is activated. However, the degree of EBV methylation in LCLs and latency III-infected BL cells is generally lower than that in type I latent-infected BL cells. Wp also maintained stable silencing and methylation in type III latent infection BL cells (Paulson and Speck 1999; Robert et al. 2020). It has been found that DNMT3b plays a critical role in Wp methylation, and the methyltransferase inhibitor 5-azacytidine (5-AzaC) can induce Wp demethylation in EBV-positive cells and reactivate the transcriptional activity of Wp. DNA methylation is the main means of regulatory “switching from Wp to Cp” in B cells after primary infection (Table 1).

**Table 1:** Methylation features of EBV gene promoters

Promoter(s)	EBV proteins	Latency phase				Lytic phase
		Cp off			Cp on	
		Latency 0	Latency I	Latency II	Latency III	
**Cp**	EBNA1, 2, 3A–C, LP	**-**	**-**	**-**	** + **	NR
**Wp**	EBNA1, 2, 3A–C, LP	**-**	** +/- **	**-**	** +/- **	NR
**Qp**	EBNA1	**-**	** + **	** + **	** + **	NR
**EBERp**	EBER1, EBER2	** + **	** + **	** + **	** + **	NR
**LMPp**	LMP1, LMP2A, LMP2B	**-**	**-**	** + **	** + **	NR
**Zp, Rp**	BZLF1, BRLF1	**-**	**-**	**-**	**-**	** + **

Cp, *BamH I C* promoter; Wp, *BamH I W* promoter; Qp, *BamH I Q* promoter; EBERp, *EBER* promoter; LMPp, *LMP* promoter; Zp, *BZLF1* promoter; Rp, *BRLF1* promoter; +, expression; -, not expressed; NR, not reported

## DNA methylation of Cp

The main transcriptional regulatory elements of Cp include the viral origin of replication OriP and EBNA2 effector elements (Li et al. 2020a; Robertson 2000). Sulfite genome sequencing and methylation-sensitive polymerase chain reaction confirmed that Cp shows extensive unmethylation in latency III infection and that all EBNAs are expressed by Cp. Cp is hypermethylated and silent in latency I and II infection (Li et al. 2021; Salamon et al. 2001). It has been shown that Wp and Cp are hypermethylated in B lymphocytes of healthy carriers (Robertson and Ambinder 1997), and no transcripts initiated by Wp and Cp are detected. *De novo* DNA methyltransferase 3b (DNMT3b) is also an important regulator of Cp methylation. David et al. found that DNMT3b was upregulated in BL cell lines that maintained latency I, while reduction of DNMT3b levels failed to induce Cp reactivation (Hughes et al. 2012). However, other research has found that treatment of BL cells with 5-azaC can lead to the activation of Cp and the expression of EBNAs (Chau and Lieberman 2004; Tempera and Lieberman 2014). Lu et al. found that the DNA demethylase TET2 cooperates with the viral oncogene EBNA2 at RBP-jκ sites to regulate the demethylation of the EBV epigenome in latency III (Lu et al. 2017). Cp methylation is important for viruses to evade immune recognition because all viral immunodominant proteins are expressed from Cp. It has been reported that cells with “Cp on” latency only survive when cytotoxic T cells targeting EBNAs are lost or defective (Niller et al. 2016). This means that activating Cp and inducing EBNAs expression through demethylating drugs may have therapeutic potential for EBV-associated diseases.

## DNA methylation of Qp

Similar to the host housekeeping gene, Qp is a promoter that is rich in CpG and lacks a TATA box and is also regulated by cellular Sp1 transcription factors (Bristol et al. 2018). Unlike other latent EBV promoters, it always remains unmethylated, regardless of its activity. During latency I, Qp transcribes EBNA1, and Qp is active and does not require methylation; in latency III (such as LCLs), the activity of Qp is silent but still not methylated (Paulson et al. 2002). It is suggested that DNA methylation is not involved in the silencing of Qp in latency III-infected cells, and the silencing may be regulated by other mechanisms. Tempera et al. found that the chromatin insulator protein CTCF prevents the encroachment of the CpG methylation at the Qp initiation site (Takacs et al. 2010; Tempera et al. 2010).

## Unmethylation of the EBER promoter

The EBER promoter is located upstream of OriP, and it is non-CpG methylated. EBERs are expressed in all latency states, and this property may help EBV drive B cell immortalization (Volk et al. 2020). The patterns of EBV genome methylation are specific and selected. Certain viral promoters, such as the Qp and EBER promoters and flanking sequences, appear to never be methylated (Takacs et al. 2010).

## DNA methylation of LMP promoters

LMP1, LMP2A, and LMP2B are three expression products of the *LMP* gene in latencies II and III. LMP1 serves as the main oncogene product in virus-infected cells and functions in tumor invasion, migration, and angiogenesis (Ai et al. 2012; Nkosi et al. 2020). LMP2A can suppress the expression of EBV lytic proteins BZLF1 and g350/220, helping EBV maintain a long-term latent infection in the host. LMP2B can regulate the distribution and function of LMP2A and jointly maintain latent EBV infection (Hino et al. 2009). LMP promoters are methylated in latency I infection. However, LMP promoters are not methylated and express LMP1 and LMP2 in latency II and III infection. LMP1 activates DNMT1 through the JNK pathway and downregulates the expression of TETs, leading to DNA methylation of host tumor suppressor genes (TSGs) in NPC. It can also increase the expression of DNMT1 by upregulating miRNA-155 (Stanland and Luftig 2020; Zhang et al. 2021). These results indicate that EBV regulates the mechanism of DNA methylation in host cells mainly through LMPs activation of DNMTs.

## Epigenetic silence of lytic promoters in latency

During latent EBV infection, lytic gene regulatory elements are usually silent. EBV lytic infection is triggered by the expression of two immediate early genes, *BZLF1* (*Zta*) and *BRLF1* (*Rta*), which are initiated by Zp and Rp, respectively (Jin et al. 2010). *Zta* can also be transcribed by Rp. *Zta* is a transcriptional activator that activates viral lytic gene expression and initiates the switch of EBV from latency to the lytic life cycle; thus, the activation of Rp triggers the lytic cascade (Germini et al. 2020). Rp and Zp are highly methylated in most latency I and III cell lines. The physiological triggering mechanism of EBV reactivation in vivo is not clear. In in vitro cell culture, EBV lytic reactivation can be triggered by a variety of drugs, such as demethylation drugs, histone deacetylase inhibitors and phorbol ester, and directly or indirectly activate *BZLF1* and *BRLF1* gene promoters, triggering lytic replication (Honeywell et al. 2018).

## BZLF1 preferentially recognizes methylated binding sites in lytic activation

Anne et al. reported that BZLF1 preferentially binds to meZRE CpG-methylated motifs in key viral promoters and overcomes heavily repressed chromatin without the need for active DNA demethylation (Woellmer et al. 2012; Woellmer and Hammerschmidt 2013). Marisa et al. reported that BZLF1 interacts with the chromatin remodeller INO80. Upon BZLF1 binding, nucleosomes are removed, Polycomb repression is lost, and RNA polymerase II is recruited to activate early promoters, promoting efficient lytic viral gene expression (Buschle and Hammerschmidt 2020; Schaeffner et al. 2019). These findings document that DNA methylation is a prerequisite for EBV lytic reactivation.

## Aberrant DNA methylation of the host genome in patients with EBV-associated diseases

### Global DNA hypermethylation and TSG silencing in EBV-associated neoplasms

After EBV infection, the EBV genome is first regulated by DNA methyltransferase methylation, which leads to extensive methylation of the virus genome and restricts the expression of lytic genes to establish latent infection (Scott 2017). Subsequently, EBV expresses a limited number of viral genes that affect the expression of host genes, including tumour suppressor genes, by regulating DNA methylation enzymes (Fiches et al. 2020). This regulation plays a central role in the maintenance of EBV latent programs in different neoplasms. EBV-regulated methylation plays an important role in EBV carcinogenesis. It forms a self-defense mechanism through its own gene methylation and induces aberrant methylation of host cell genes, leading to the cell cycle disorder and cell transformation and promoting the occurrence and development of EBV-associated neoplasms through epigenetic mechanisms.

### EBVaGC

According to the Cancer Genome Atlas (TCGA) classification of gastric cancer, EBVaGC has unique molecular characteristics compared with EBV-negative gastric carcinoma (EBVnGC), such as *PIK3CA* mutations, DNA hypermethylation, *ARID1A* and *BCOR* mutations, and JAK and PD-L1/2 amplifications (Zheng et al. 2020). Liang et al. identified 216 genes downregulated by EBV-induced host DNA hypermethylation in AGS-EBV cells through epigenome and transcriptome sequencing (Liang et al. 2014). Among these genes, *CDKN2A* is a tumor suppressor that blocks the progression of the cell cycle (Saito et al. 2013), and its silencing is related to a poor prognosis and survival rate (Shi et al. 2012). Another tumor suppressor, *FHIT*, which is implicated in tumour growth suppression, as well as in the induction of apoptosis, also has obvious methylation in EBVaGC (He et al. 2015). The promoter of *RCOR2* is also methylated in EBVaGC tissue; RCOR2 is mainly highly expressed in embryonic stem cells, and its encoded protein interacts with the histone demethylase LSD1 (Yang et al. 2011). A recent study showed that the tumor suppressor *CYLD*, which encodes a multifunctional deubiquitinase and negatively regulates multiple signal transduction pathways, is hypermethylated and has decreased expression in EBV-associated gastric adenocarcinoma (Ghadami et al. 2019). In addition, some genes, such as *E-cadherin*, *CTNNBIP1*, and *PTEN*, which are involved in cancer-related cell adhesion, cell migration, the WNT pathway, and the MAPK signaling pathway, have also been found to be hypermethylated in EBVaGC (Kosari-Monfared et al. 2019; Yang et al. 2020). EBVaGC is intermediate between latency I and II and mainly expresses EBERs, EBNA1, and BARTs with almost no or low LMP1 and LMP2B. LMP2A is expressed in approximately 40% of patients (Strong et al. 2013). It has been shown that LMP2A upregulates the expression of the methyltransferase DNMT1 and downregulates the expression of the key demethylase TET2 (Fiches et al. 2020). LMP2A enhances the expression of DNMT1 by inducing phosphorylation of STAT3, resulting in *PTEN* silencing through hypermethylation of the promoter in an IL-6-independent manner (Wu et al. 2017). It can also induce ERK phosphorylation to upregulate DNMT3a, leading to methylation of the water-glycerol channel protein AQP3 promoter and resulting in downregulation of AQP3 (Wang et al. 2019). LMP1 is expressed at low levels in EBVaGC, and only 10% of patients express LMP1 (Yang et al. 2020). Li et al. concluded that all LMP promoters were methylated to different degrees and that the expression of LMP1 could be recovered using a demethylation agent (Li et al. 2016). Gao et al. found that LMP1 leads to hypermethylation of the tumour suppressor gene *RASSF10* by upregulating the expression of DNMT1. *RASSF10* encodes a protein that inhibits cell proliferation, invasion, and migration and induces apoptosis. Overexpression of LMP1 in human gastric adenocarcinoma AGS cells promotes cell migration, invasion, and colony formation; when RASSF10 is coexpressed, this effect is counteracted (Gao et al. 2021). Currently, the mechanism by which EBV regulates DNA methylation in GC is still unknown. However, the knowledge summarized at present shows that EBV regulates the mechanism of DNA methylation in host cells mainly through LMP1/2A activation of DNMT1/3 and inhibition of TET2 (Table 2) (Okabe et al. 2017).

**Table 2:** Methylation features in EBV-associated diseases

Cancer	Latency program	Virus protein	Signal pathway	DNA methyltransferase	Effect	Ref
EBVaGC	Between I and II (> 40% express LMP2A)	LMP2A	Phosphorylates STAT3	DNMT1↑	*PTEN* hypermethylation	(Hino et al. 2009)
			Phosphorylates ERK	DNMT3a↑	*AQP3* hypermethylation	(Wang et al. 2019)
			NR	TET2↓	NR	(Namba-Fukuyo et al. 2016)
		LMP1	NR	DNMT1↑	*RASSF10* hypermethylation	(Gao et al. 2021)
NPC	II	LMP1	Activates JNK/AP-1 signalling pathway	DNMT1↑	*E-cadherin* promoter silencing, increases proliferation, invasion, metastasis	(Tsai et al. 2006) (Tsai et al. 2002)
			Activates NFκB signalling pathway	DNMT3b↑	*PTEN* hypermethylation	(Peng et al. 2016)
			Activates miRNA-155	DNMT1↑	TSG promoter silencing	(Lu et al. 2008)
		LMP2A	NR	NR	*S100A4* promoter demethylated, increases invasion and metastasis	(Lin et al. 2016)
BC	NR	NR	NR	NR	*BRCA1/2*, *p14*, *p16*, and *hMLH* hypermethylation	(Yahia et al. 2014) (Abdallah et al. 2018)

EBVaGC, EBV-associated gastric carcinoma; NPC, nasopharyngeal carcinoma; BC, breast cancer; ↑, upregulation; ↓, downregulation; NR, not reported

### EBV-associated NPC

EBV is closely associated with the development of NPC. EBV establishes latency II infection in EBV-associated NPC. The main viral products expressed in NPC are EBNA1, LMP1, and LMP2A, and the CpG sites of LMP are hypermethylated (Ernberg et al. 1989). This methylation is different from silencing of TSG expression and helps EBV evade the surveillance of the immune system; thus, EBV genome methylation plays an indispensable role in maintaining the latent infection state of EBV in NPC (Allday et al. 1990; Lam et al. 2019). It has been found that the YYD domain of COOH terminal activation region 2 (CTAR2) of LMP1 in NPC cells is the key to activating the JNK signaling pathway, which leads to c-Jun phosphorylation. c-Jun (homodimer) or c-Fox (heterodimer) forms AP-1 (Fos/Jun) complexes, which bind to the DNMT1 promoter region and enhance the expression of DNMT1 (Tsai et al. 2006). Tsai et al. confirmed that LMP1 induces DNMT1 to increase the methylation level of *E-cadherin* and inhibit its expression by activating the JNK/AP-1 signalling pathway (Tsai et al. 2002). As a result, the invasion and metastasis of NPC are regulated. LMP1 can also activate DNMT3b through the NFκB signaling pathway (Ammous-Boukhris et al. 2019; Peng et al. 2016) and activate DNMT1 through miRNA-155, thereby inhibiting TSGs in a DNA methylation-dependent manner (Lu et al. 2008). Recent studies have found that positive expression of LMP1 is significantly correlated with a poor overall survival rate (Shi et al. 2019). In addition, DNA hypomethylation of *S100A4* was found in LMP2A-positive NPC tissues. S100A4 can enhance the invasion and metastasis of NPC cells in vitro and in vivo, and researchers found that inhibition of DNA methyltransferase by 5-aza-DC stimulated the expression of S100A4 in cells without ectopically expressing LMP2A. Methylation-specific PCR (MSP) confirmed that ectopic expression of LMP2A led to demethylation of the *S100A4* promoter. These results suggest that LMP2A-induced hypomethylation is involved in the regulation of S100A4 expression in NPC (Table 2) (Lin et al. 2016). Consequently, hypomethylation and activation of related genes are also important for the progression of cancer metastasis. Analysis of the epigenetic modification of EBV-associated tumours can help elucidate the pathogenesis of NPC and promote the development of novel treatments (Niller et al. 2009).

### EBV-associated HL

Fifty percent of HL is associated with EBV infection (Bu et al. 2019). EBV establishes latency II infection in EBV-associated HL. The main expressed products are EBNA1 and LMP1 (Zhou et al. 2015). EBV Cp can evoke the expression of virus proteins targeting CD8^+^ cytotoxic T cells, but these proteins are generally not expressed in EBV-related HL. It has been found that EBV can mask the presence of the virus in tumor tissue by inhibiting the expression of virus protein antigen and methylation of transcriptional control sequence, escaping the immune surveillance of CD8^+^ cytotoxic T cells and promoting tumorigenesis (Dhiab et al. 2015; Kis et al. 2011). Myriam et al. found that tumor suppressor gene (*p16*, *RASSF1A*, *CDH1*, *DAPK*, *GSTP1*, *SHP1*, and *MGMT*) promoter methylation occurs in both EBV-positive and EBV-negative HL. Surprisingly, most of these gene promoters are more frequently hypermethylated in EBV-negative HL cases than in EBV-positive HL cases. In particular, the *DAPK* gene promoter had higher methylation levels in LMP1-negative cases than in LMP1-positive cases (Dhiab et al. 2015). This suggests that *DAPK* gene promoter hypermethylation may represent an alternative mechanism for the survival of neoplastic B cells in the absence of EBV infection. These observations likely reflect the multitude of factors involved in HL development and the complexity of their interactions with epigenetic factors.

### EBV-associated BL

BL tumours are most frequently associated with EBV infection, as 15–85% of BL shows EBV infection (Hammerl et al. 2019). EBV replicates in latency I in EBV-associated BL and mainly expresses EBNA1. It often shows epigenetic changes, and the viral genomes Cp and Wp are hypermethylated and inactivated. Qp plays a role in maintaining the unmethylated state and directs the transcription of EBNA1. Tao et al. used bisulfite genome sequencing to detect the methylation of frozen endemic BL tissue sections and found no methylation of Qp but complete methylation of Cp in EBNA1(Tao et al. 1998; Zheng et al. 2020). Paschos et al. suggested that the epigenetic mechanism of BL is related to the downregulation of Bim, a member of the apoptotic Bcl-2 family. EBV triggers a series of events to suppress the epigenetics of the tumor suppressor gene *Bim* in infected B cells and inhibit their passage (Paschos et al. 2009).

**Fig. 2:**
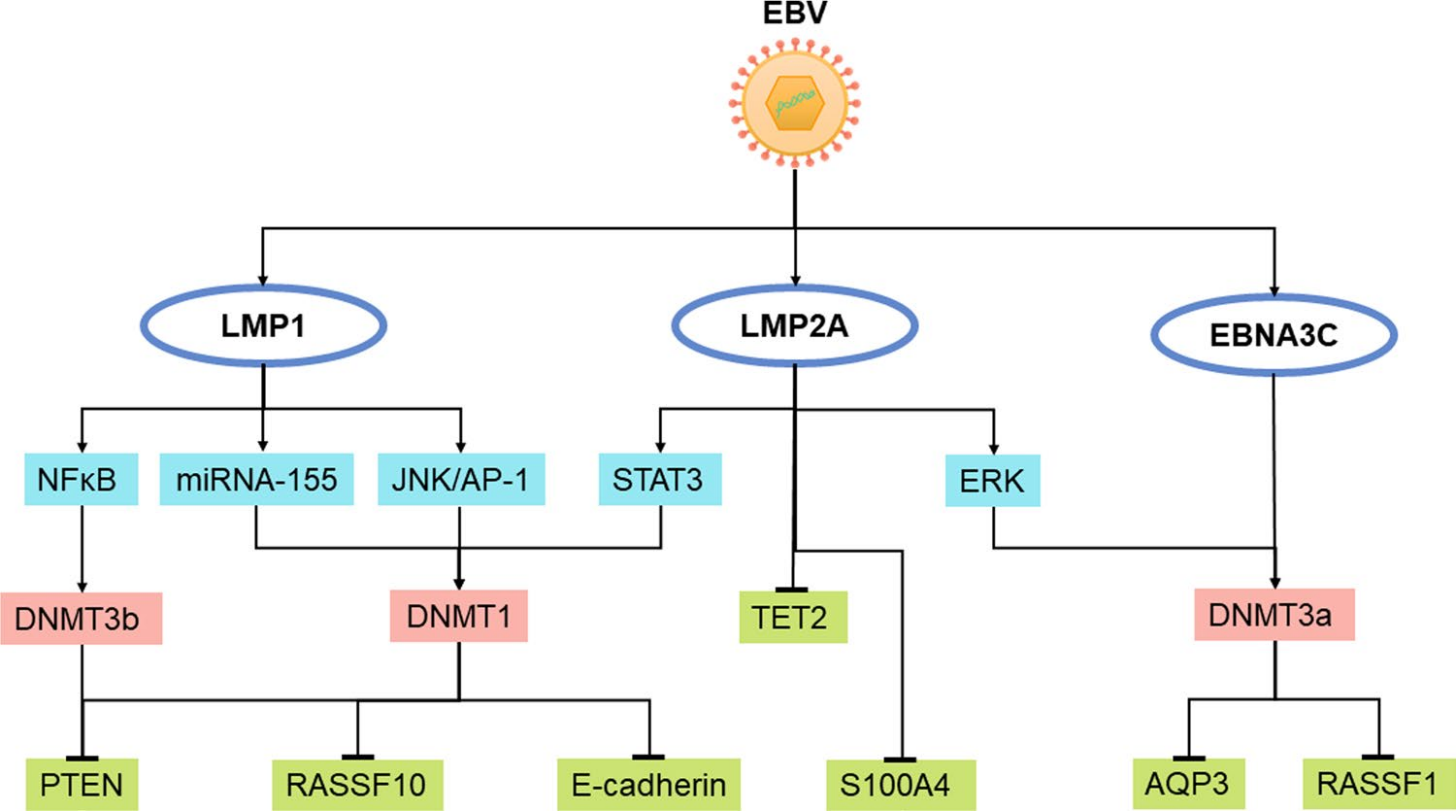
Schematic presentation of EBV regulation of host genome methylation in EBV-associated neoplasms

Some studies have documented that hypomethylation of certain promoter regions occurs in the early stages of EBV-mediated transformation from resting B cells (RBLs) to proliferating LCLs (Hernando et al. 2013). Further analysis of these hypomethylated regions showed enrichment of specific transcription factors, such as the NFκB/p65 pathway, and high expression of these genes. These results indicate that hypomethylation related to EBV-mediated transformation of RBLs to LCLs is related to proliferation. Hagman et al. also found significantly increased expression of the transcription factors EBF1, IRF4, and MEF2C, and EBF1 plays an essential role in B lymphocyte production and B cell function (Hagman et al. 2012). IRF4 is a key transcription factor for the production of normal plasma B cells (Ikeda and Tagawa 2021). MEF2C is necessary for the proliferation and survival of B cells after antigen receptor stimulation (Wang et al. 2020). Additionally, drug-induced demethylation improved the transformation efficiency and proliferation ability of resting B cells to lymphoblasts. EBV regulates methylation of the NFκB pathway, which plays an important role in EBV infection as well as in the occurrence and development of malignant tumours (Tempera and Lieberman 2014). A recent study found that overexpression of EBNA3C in the EBV-negative BL cell line BJAB can induce DNMT1 protein expression, and EBNA3C can interact with DNMT1 (Pandey and Robertson 2018). Another study found that overexpression of EBNA3C in LCLs could induce the expression of DNMT1 and DNMT3a, which further shows that the promoter of TSG *RASSF1* was hypermethylated in BJAB and LCLs overexpressing EBNA3C, suggesting that EBNA3C could downregulate the expression of RASSF1 by enhancing the methylation activity of DNMTs (Fig. 2) (Zhang et al. 2019). Taken together, it seems that EBV can regulate the expression of host genes through methylation mechanisms in order to promote transformation and proliferation of BL.

### EBV-associated BC

Some studies have shown that some BC is associated with EBV infection, and its epigenetic characteristics are characterized by the low frequency of oncogene mutations and the high frequency of epigenetic silencing of BC-related TSGs (Yahia et al. 2014). Through genomic DNA methylation sequencing analysis of EBV-associated BC tissues, Mohammad et al. found that key TSGs of the host genome were hypermethylated, such as *BRCA1/2*, *p14*, *p16*, and *hMLH* (Table 2) (Yahia et al. 2014). In addition, the main developmental pathways, including the Hippo signaling pathway, are also highly methylated (Abdallah et al. 2018). However, the mechanism of the role of EBV proteins in regulating the methylation of BC requires further investigation.

## EBV-associated nonneoplastic diseases

Currently, epigenetic modification research on EBV infection is mostly focused on EBV-associated tumours, and there are very few studies on EBV infection**-**associated nontumor diseases. In particular, the mechanism underlying the regulatory effect of EBV protein on host genome DNA methylation in these diseases has not been reported.

### IM

IM is a self-limited lymphoproliferative disease caused by EBV infection. Latency III is characteristic of IM, and EBV expresses all latent genes. Tierney et al. found that in most IM patients, Wp was hypermethylated and Cp was nonmethylated (Tierney et al. 2000). Cp activity is the main difference between latency III and other latent forms. However, the role of DNA methylation in the development of IM, especially at specific genomic regions, such as host gene promoter CpG islands, has not yet been deeply investigated and warrants further research.

### CAEBV

CAEBV is a nonneoplastic lymphoproliferative disease associated with EBV infection. Patients with CAEBV have a poor prognosis, and its pathogenesis and the role of EBV are unclear. CAEBV is a type II latency disease in which only EBNA1 is expressed in EBNAs. Yoshioka et al. detected DNA methylation in spleen tissues of five patients with CAEBV and found that the Cp and Wp promoters were hypermethylated and that Qp was not methylated (Yoshioka et al. 2003). However, they did not find the Qp transcript. Surprisingly, EBNA1 was transcribed by Cp in CAEBV samples. These data suggest that the hypermethylation of the Cp region may not be sufficient to prevent the transcription of Cp, and the transcripts initiated by Cp may have a unique splicing mode and prioritize the induction of transcriptional EBNA1 in most CAEBV cases, but its mechanism needs to be explored further. The seemingly contradictory and unique relationship between the hypermethylation of the CAEBV Cp region and the transcriptional activation of Cp provides an idea for further study of the pathogenic mechanism of CAEBV.

## Application of methylation modification in the diagnosis and treatment of EBV-associated diseases

### Early diagnosis and prognosis assessment

Hypermethylation of DNA in the peripheral blood provides new insight into the early diagnosis and screening of tumors. Dying cells release DNA fragments into the bloodstream, and this plasma-derived circulating cell-free DNA (cfDNA), also known as “liquid biopsy”, is a promising tool for minimally invasive molecular diagnostics and disease monitoring (Dor and Cedar 2018). cfDNA can be used to track cancer progression or response to treatment even if the tumour is inaccessible or its location is unknown. Some studies have reported that methylation of the promoter of the tumour suppressor genes *RUNX3*, *RASSF1A*, and *Reprimo* may be a powerful and potential noninvasive biomarker for the detection and diagnosis of gastric cancer (Wen et al. 2017). Promoter methylation of *p16* can often be detected in tumor samples but not in normal tissues. Therefore, the detection of serum *p16* methylation may be a useful marker of early gastric cancer (Liu et al. 2019). Plasma EBV DNA methylation detection has important potential in the diagnosis of NPC (Lam et al. 2019). Therefore, DNA methylation sequencing may be used as a new method for early and noninvasive tumour diagnosis. Moreover, methylated cancer-specific changes can also provide a sensitive indicator of severity and an auxiliary marker of prognosis (Kim et al. 2014). However, the DNA of circulating tumor cells may not be completely released into the blood, and the proportion of cfDNA in the total blood DNA varies widely from very rare (0.01%) to highly prevalent (> 90%) and is histology- and tumor burden-dependent (Liu et al. 2021). Therefore, it has become an obstacle to reliably identifying the methylation level of cfDNA in the blood. Nevertheless, whether “fluid biopsy” could provide sufficient sensitivity and specificity for clinical diagnosis and whether it could correctly distinguish patients with cancer remain unconfirmed.

## Treatment

DNA demethylation is promising as a new target for tumor therapy. The genetic damage of tumors with DNA methylation is easier to correct than that of tumors with DNA sequence mutation because of the reversibility of epigenetic modification. This concept has led to the development of drugs that are useful in the treatment of specific tumors (Jones et al. 2016). Although DNA methylation may not play a predominant role in all cancers, the modification patterns will affect cell tendency and tumor phenotype to varying degrees (Dor and Cedar 2018). In cancer treatment, drugs such as azacytidine administered alone or in combination with other compounds could cause global demethylation among target cells (Pulecio et al. 2017). 5-AzaC, a demethylating drug, mainly binds to DNA methyltransferase through covalent bonds to form an irreversible complex, which reduces the level of genomic methylation and reactivates hypermethylated tumour suppressor genes. At present, many studies have confirmed that most of the CpG islands in the promoter region of many tumour suppressor genes are in a state of hypermethylation, and the expression of protein can be restored after intervention with the demethylated drug 5-AzaC (Sun et al. 2020). Additionally, it has been reported that zebularine can activate the tumor suppressor genes *p15*, *p16*, and *p57* by demethylation in gastric cancer cells (Pan et al. 2016). Moreover, zebularine treatment sensitized the cGAS-STING pathway to recruit CD8^+^ T cells and NK cells into the tumor microenvironment by demethylating the *STING* promoter (Lai et al. 2021). However, demethylating drugs not only restore the expression of tumor suppressor genes but also affect the expression of oncogenes. Therefore, the pros and cons of demethylating drugs warrant further investigation.

## Conclusion and prospects

Selective activation of EBV promoters is regulated by DNA methylation. All promoters are nonmethylated upon infection, and the *EBNA* genes are transcribed via Wp. Wp becomes inactive due to DNA methylation shortly after infection. Cp shows extensive unmethylation in latency III infection, and all *EBNAs* are transcribed by Cp. However, Cp is hypermethylated and silent in latency I and II infection, Qp is active, and only EBNA1 is expressed during latency I. The silencing of the EBV genome facilitates immune evasion in latently infected cells through DNA methylation, while Qp remains unmethylated to support the latent state, which is probably also supported by the binding of chromatin insulator protein CTCF that prevents the encroachment of CpG methylation at the Qp initiation site. Moreover, DNA methylation is a prerequisite for escape from viral latency, and the immediate early protein BZLF1 preferentially binds to CpG-methylated motifs meZRE in key viral promoters to promote efficient lytic viral gene expression without the need for active DNA demethylation. Thus, EBV regulates its latent infection and reactivation with the help of cell-mediated DNA methylation.

Epigenetic markers and differentially methylated genes may have diagnostic and prognostic value in EBV-related diseases. EBV infection results in hypermethylation of EBV promoters and the host genome in NPC and EBVaGC, and is associated with downregulation of TSGs. Due to the reversibility of epigenetics, drugs that affect the epigenetic characteristics of EBV-associated tumors may be developed for treatment. Hypomethylating agents have demonstrated the potential to drive hypomethylation of the viral genome and initiate re-expression of immunogenic viral genes in EBV-associated lymphoma cells. Therefore, targeting DNA methylation is a potential novel therapeutic strategy for EBV-associated diseases.
