# Correction to: The roles of DNA methylation on the promotor of the Epstein–Barr virus (EBV) gene and the genome in patients with EBV‑associated diseases

**DOI:** 10.1007/s00253-022-12216-2

**Published:** 2022-10-07

**Authors:** Linlin Zhang, Ran Wang, Zhengde Xie

**Affiliations:** 1 Present Address: Beijing Key Laboratory of Pediatric Respiratory Infectious Diseases, Key Laboratory of Major Diseases in Children, Ministry of Education, Nationa Clinical Research Center for Respiratory Diseases, Beijing Pediatric Research Institute, Beijing Children’s Hospital, Capital Medical University, National Center for Children’s Health, Beijing, 100045 China; 2 Research Unit of Critical Infection in Children, 2019RU016, Chinese Academy of Medical Sciences, Beijing, China

**Correction to: Applied Microbiology and Biotechnology (2022) 106:4413–4426**

**https://doi.org/10.1007/s00253-022-12029-3**

In the published version, affiliation 2 and Fig. 2 have errors.

The correct details for affiliation 2 should be as below.

Research Unit of Critical Infection in Children, 2019RU016, Chinese Academy of Medical Sciences, Beijing, China

The correct image for Fig. 2 is shown below.

**Fig. 2:**
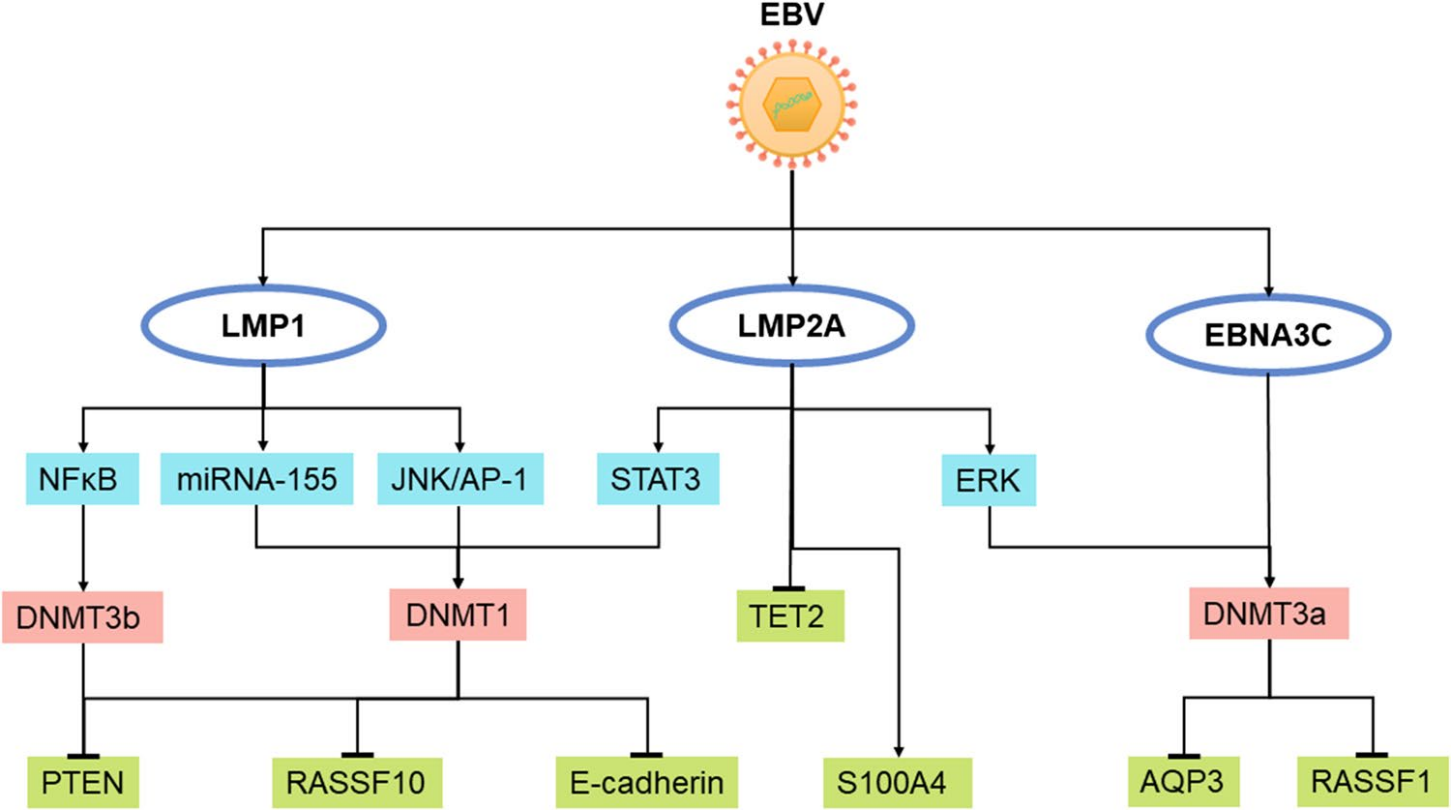
Schematic presentation of EBV regulation of host genome methylation in EBV-associated neoplasms

